# SPTEdb: a database for transposable elements in salicaceous plants

**DOI:** 10.1093/database/bay024

**Published:** 2018-03-09

**Authors:** Fei Yi, Zirui Jia, Yao Xiao, Wenjun Ma, Junhui Wang

**Affiliations:** 1 State Key Laboratory of Tree Genetics and Breeding, Key Laboratory of Tree Breeding and Cultivation of State Forestry Administration, Research Institute of Forestry, Chinese Academy of Forestry, Beijing 100091, China and; 2 College of Biological and Pharmaceutical Sciences, Three Gorges University, Yichang 443002, China

## Abstract

Although transposable elements (TEs) play significant roles in structural, functional and evolutionary dynamics of the salicaceous plants genome and the accurate identification, definition and classification of TEs are still inadequate. In this study, we identified 18 393 TEs from *Populus trichocarpa*, *Populus euphratica* and *Salix suchowensis* using a combination of signature-based, similarity-based and *De novo* method, and annotated them into 1621 families. A comprehensive and user-friendly web-based database, SPTEdb, was constructed and served for researchers. SPTEdb enables users to browse, retrieve and download the TEs sequences from the database. Meanwhile, several analysis tools, including BLAST, HMMER, GetORF and Cut sequence, were also integrated into SPTEdb to help users to mine the TEs data easily and effectively. In summary, SPTEdb will facilitate the study of TEs biology and functional genomics in salicaceous plants.

**Database URL**: http://genedenovoweb.ticp.net:81/SPTEdb/index.php

## Introdution

Transposable elements (TEs) have the ability to move throughout genome and insert themselves into new locations. They are ubiquitous in higher eukaryotes and represent a significant fraction of the genomes, particularly of plant genomes (1). For example, approximately 40% of the rice genome (2) and 80% of the wheat genome (3) are estimated to be TEs, respectively. Based on the transposition mechanisms, TEs are classified as retrotransposons (class I) and DNA transposons (class II). Retrotransposons transpose via RNA intermediate by ‘copy and paste’ mechanism, whereas class II TEs move through a direct ‘cut and paste’ mechanism (4). Within each of these classes, TEs are further subdivided based on the structural features of their sequences. Class I TEs are grouped into five orders, long terminal repeat (LTR) retrotransposons, *DIRS*-like elements, *Penelope*-like elements (PLEs), LINEs and SINEs (4). Class II TEs are classified into four main orders, terminal inverted repeats (TIRs), *Crypton*, *Maverick* and *Helitron* (4, 5).

In contrast to be portrayed as ‘junk DNA’ or ‘selfish elements’, emerging evidence suggested that TEs contribute to chromosome structure (6), genome size (7), genome rearrangement (8), gene creation (8) and gene expression and regulation (9). For instance, recent study suggested that TEs (MITEs) may exert a general regulatory function at translational level (10). Meanwhile, transposon is a huge challenge (11) for genome sequencing (12), assembly (13) and annotation (14) due to its repetitive feature. Therefore, the precise identification, classification and annotation of TEs at the whole genome level are very important (15).

*Salicaceae* consists of 650 species in the world and is divided into three genera, namely *Chosenia* Nakai, *Populus* L. and *Salix* L (16). Sequencing salicaceous genomes is relevant because of their ecological significance and economic importance. Three salicaceous genomes, *Populus trichocarpa* (17), *Populus euphratica* (18) and *Salix suchowensis* (19), have been sequenced with the development of advanced sequencing technologies. At present, researchers can obtain the TEs information of *Salicaceae* from some genome or repeat databases, such as PtGDB and PGSB-REcat. But the TE annotation of these genomes is incomplete and is based on different methods. In this work, TEs in the genomes of sequenced salicaceous plants were identified, classified and annotated by a combined approach. We organized the obtained TEs into a salicaceous plants TEs database, SPTEdb. Many tools and other databases were integrated into SPTEdb to facilitate the study of users. As such, SPTEdb provides a platform to study TEs biology and functional genomics in salicaceous plants.

## Construction and content

### System implementation

The sever of SPTEdb was constructed using Linux Ubuntu 12.04, Apache 2, MySQL Server 5.5 and Perl 5.16.3/PHP 5.3. All TEs data and information were stored in MySQL tables for quick response and efficient management. The CGI programs were mainly developed using JavaScript, Perl and PHP programming languages. The JBrowse Genome Browser (version 1.12.0), a genome browser built with HTML5 and JavaScript, was used for manipulation and displays the genome coordinates of TEs in the three salicaceous plants in SPTEdb (20).

### Data sources

The download address for the genome sequences of three salicaceous plants are listed in Table 1.

**Table 1.** List of salicaceous plant species analyzed in this study

Plant species	URL
*Populus trichocarpa*	ftp://ftp.ncbi.nlm.nih.gov/genomes/all/GCF/000/002/775/GCF_000002775.3_Poptr2_0/GCF_00000277
*Populus euphratica*	ftp://ftp.ncbi.nlm.nih.gov/genomes/all/GCA/000/411/955/GCA_000411955.5_PG29_v4.1/GCA_000411955.5
*Salix suchowensis*	http://115.29.234.170/blast/db/Willow.fa.tar

### Identification of TEs in the three salicaceous plants

In order to make a complete and accurate identification of TEs in the three salicaceous plants, a combination of multiple methods were employed, including signature-based, similarity-based and *de novo* methods (11). The identification process of three salicaceous plants is identical, so *P.trichocarpa* is taken as an example to illustrate the process.

Step 1: Identification of TEs using signature-based tools. LTR_FINDER (version 1.05) (21) and MGEScan-nonLTR (version 2.0) (22) programs were used with default parameters to search against the genome of *P.trichocarpa* for identification retrotransposons, then obtained 10 131 sequences and 74 sequences, respectively. There were 78 MITE transposons been detected using MITE-Hunter (version 20100819) (23) with default parameters. For Helitron transposons, HelitronScanner (version 1.1) (24) was employed with default parameters and got 1340 sequences. A total of 11 623 transposons were identified using the approaches above.

Step 2: Identification of TEs using similarity-based tools. Using RepeatMasker (version open-4.0, default parameters, http://www.repeatmasker.org), the genome of *P.trichocarpa* was searched against Repbase database (version 20150723, http://www.girinst.org/repbase/) (25, 26) for further similarity-based identification of TEs. The results were filtered in line with the criterion that those scores <250 or target coverage <40% were removed. We extracted 31 TIR transposons from the abovem-entioned results according to the characteristics of conservative domain in DNA transposons.

Step 3: Identification of TEs using *de novo* tools. For *de novo* identification of TEs, RepeatScout (version 1.0.5) (27), PILER (version 1.0) (28) and RepeatModeler (version 1.0.7, http://www.repeatmasker.org/RepeatModeler.html) were performed with default parameters to analyze the genome of *P.trichocarpa*. The putative transposons that have >90% sequence similarity to each other were removed. In order to reduce the redundancy, the putative TEs with >90% sequence similarity to the predictions obtained from above two steps were discarded. Finally, 20 sequences were acquired from these operations.

Step 4: All the transposons obtained from steps 1 to 3 were integrated into a library for definition and classification of TEs. There were 11 674 putative TEs in the library for *P.trichocarpa*.

### Annotation of TEs in the three salicaceous plants

There are a number of different criteria for the classification and definition of TEs due to their complicated structure (4, 29, 30). In this study, we adopted the criteria proposed by Wicker et al. (4). It is a practical method and easy to learn for the researchers.

The putative TEs in the library obtained previously were compared with Repbase database using RepeatMasker (version open-4.0, default parameters), and the best hit target sequence was selected as the superfamily of putative TEs.

The superfamilies annotated earlier were subdivided into families, which were defined by DNA sequence conservation. The definition of families was performed using the 80-80-80 rule (4). Thus, two elements belonged to the same family if they shared at least 80% of sequence identity in at least 80% of their coding or internal domain, or within their terminal repeat region, or in both. Meanwhile, in order to prevent misclassification of short and possibly random stretches of homologous sequences, the shortest sequence should be longer than 80 bp.

In order to exclude the false positive, the TEs sequences of those superfamilies with <3 families in SPTEdb were extracted as query sequences, and Blastn (1e-5) was performed on the query and Repbase database (subject). In the optimal alignment, the query sequences with coverage below 80% were discarded. Such as, seven putative TEs of *P.trichocarpa* were excluded, and the remaining 11 667 TEs were divided into 543 families.

## Results

### Identification of TEs in the three salicaceous plants

A total of 18 393 TEs belonging to 1621 families were identified in the three salicaceous plant genomes, and the complete result is presented in Table 2. These information were organized into a user-friendly web-based database, SPTEdb. Compared with *P.trichocarpa*, the TEs identified in *P.euphratica* and *S.suchowensis* are much less, which were 3961 and 2765, respectively. However, the number of TEs families was only slightly different in the three plants, which were 543, 550 and 528 respectively. Two types of transposons were identified in the three plants. Retrotransposons were more abundant than DNA transposons clearly. The proportion of retrotransposons and DNA transposons in the three plants were 63.86% to 36.14%, 87.65% to 12.35%, and 54.07% to 45.93%, respectively. It was important to note that some TEs were not accurate annotated (unknown) in SPTEdb, and the lack of accurate annotation (2770 TEs) was distinct in *P.trichocarpa* especially.

**Table 2.** Summary of identified TEs in three salicaceous plants genomes

Class	Order	Superfamily	*P. trichocarpa* members/families	*P. euphratica* members/families	*S. suchowensis* members/families
Retrotransposons	LTR	*Caulimovirus*	6/5	3/3	
		*Copia*	1557/60	497/53	819/37
		*DIRS*	5/5	3/3	
		*ERV1*	52/18	28/8	3/3
		*ERVK*	10/8	9/8	4/4
		*Gypsy*	5587/39	2610/45	414/41
		*Ngaro*	6/4		
		*Pao*	140/8	10/8	5/5
		Unknown		200/139	169/104
	LINE	*L1*	87/19	112/17	81/22
Subtotal			7450/166	3472/284	1495/216
DNA transposons	TIR	*CMC*	3/1	7/6	5/5
		*hAT*	17/14	15/12	8/8
		*MuLE*	6/5	3/3	
		*PIF-Harbinger*	3/3	6/5	4/4
		*TcMar*		6/6	
		Unknown	2770/136		
	MITE	*MITE*	78/41	18/18	18/16
	Helitron	*Helitron*	1340/177	434/216	1235/279
Subtotal			4217/377	489/266	1270/312
Total			11 667/543	3961/550	2765/528

To test the reliability of our method for identifying transposons, the TEs of *Arabidopsis thaliana* were used as an example to verify. Using our method, 986 TEs were predicted (query), on the other side, 524 TEs were obtained from the Repbase database (subject). Blastn (1e-5) was performed on query and subject, and the result showed that there were 764 sequences (77.48%) in query matched on 449 sequences (85.69%) in subject. There are 271 alignments with over 80% length coverage for both query and subject (252 query matches to 169 subject).

### Web interface

**Figure 1.**
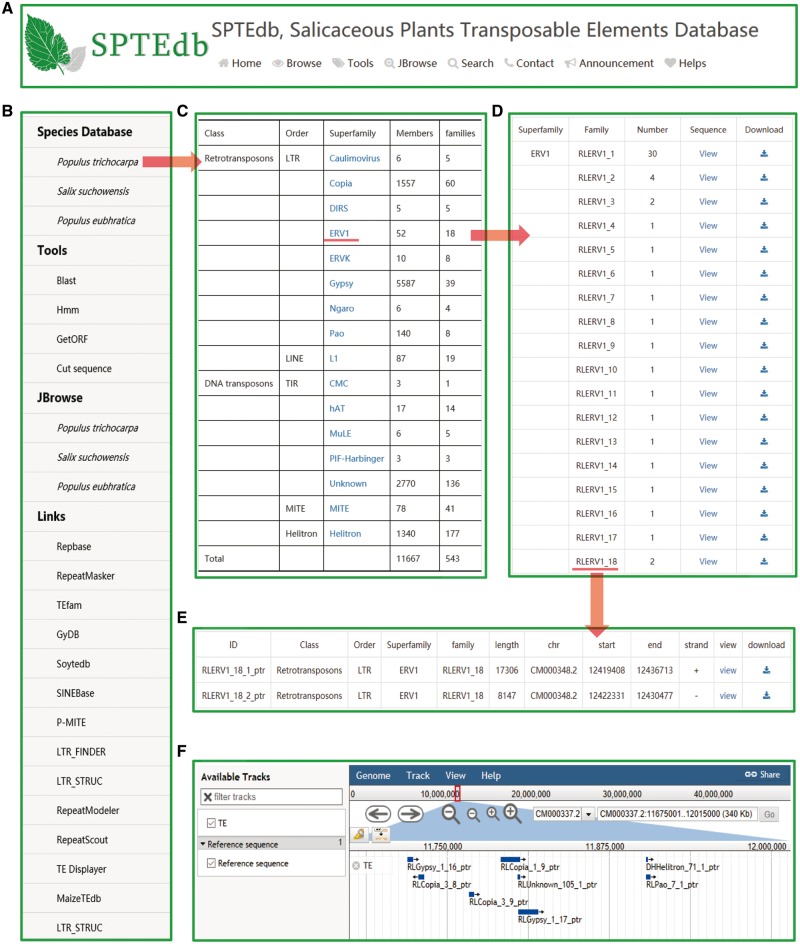
SPTEdb organization and the description of browse functional sections in the database. (**A**) The top menu of SPTEdb. (**B**) The side menu of SPTEdb. (**C**)–(**E**) The user interface of browsing in SPTEdb and the results of some samples. (**F**) Genome sequence view in JBrowse.

The SPTEdb web interface was organized into functional sections so as to provide an efficient platform to study TEs in salicaceous plants. Users can obtain the basic information about SPTEdb on the homepage. Navigation tabs are set on the top menu (Figure 1A) and side menu (Figure 1B), then each of the main navigation tab provides a specific capability for browsing, retrieving or downloading information of TEs in the database. In addition, many powerful analysis tools are supplied for the users, such as Blast, HMMER, GetORF and Cut sequence (Figure 1B).

### Browse and JBrowse

By clicking the entry of ‘Browse’ in the top menu or ‘Species Database’ in the side menu, users can acquire the information of TEs. Through the hyperlink of a selected plant species, the summary of TEs information in the form of table is provided to users (Figure 1C). Researchers can obtain detailed information of each superfamily in the ‘statistical information’ page. If the users are interested in a family, they can get the further information by clicking the corresponding entry of it (Figure 1D). Finally, the exhaustive information of every member of a family are displayed in the corresponding page, including ID, classification, length, location and sequence (Figure 1E).

JBrowse is a fast, embeddable genome browser built completely with JavaScript and HTML5, with optional run-once data formatting tools written in Perl (20). As a browse tool, graphic visualization is the most prominent advantage, and users can conveniently view the elaborate information of TEs by simply clicking the name of the TE in the graphic interface (Figure 1F).

### Search

**Figure 2.**
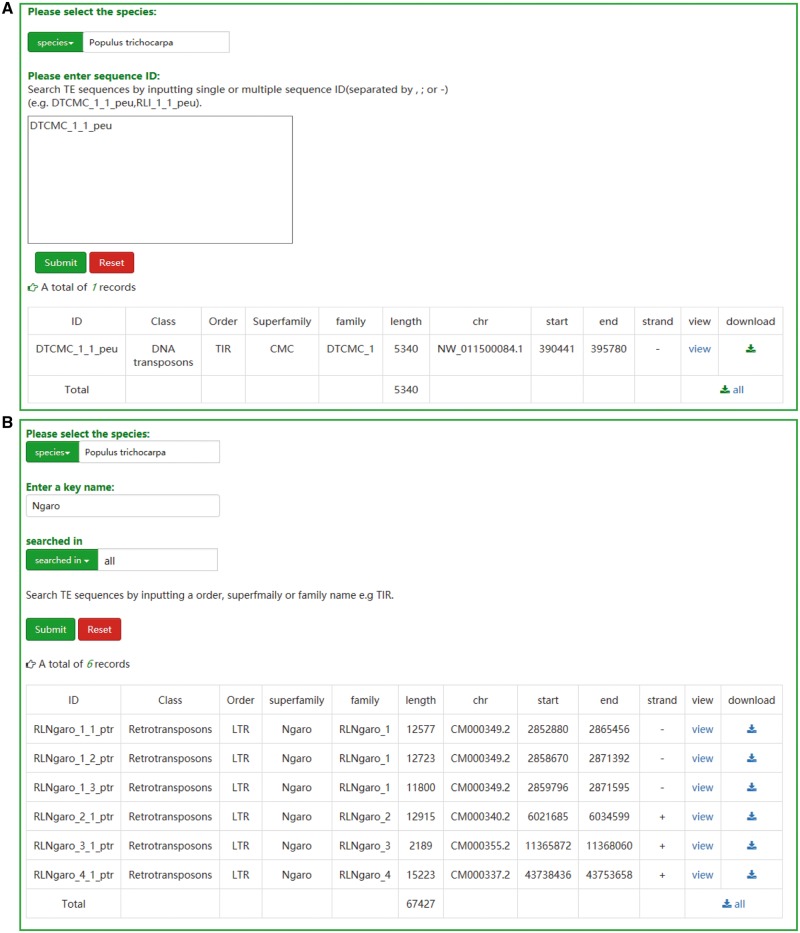
The user interface of searching in SPTEdb. (**A**) The interface of ‘search by ID’ and the result of a sample. (**B**) The interface of ‘search by family’ and the result of a sample.

Two retrieval methods, namely ‘search by ID’ and ‘search by family’, are offered to the users. If the ID of a specific transposon is known, users can search the database and acquire the relevant entry and the result will be exclusive (Figure 2A). A keyword, such as an order, superfamily or family name of TEs, is necessary for the ‘search by family’ method. In contrast to the first method, the result of ‘search by family’ is usually not exclusive, but all the TEs that contain the keyword will be displayed in a tabular format (Figure 2B). Sequences of corresponding results from two approaches can be downloaded as txt format. Furthermore, the TEs sequences can also be downloaded in browse page or JBrowse.

### Tools

To facilitate the study of users, four analysis tools were embedded into SPTEdb (Figure 1B). BLAST is a powerful and widely used sequence alignment tool, and users can submit the query sequences to do BLASTN or tBLASTN against the database for homology search. Using GetORF, the potential open reading frame (ORF) of query sequences can be analyzed. The output is a sequence file containing predicted ORFs longer than the minimum size, which is defaulted to 30 bases. HMMER is a free and commonly used software package for sequence analysis, and its general usage is to identify homologous protein or nucleotide sequences (31). As for Cut sequence, it is a tool to extract the sequence in a specified location defined by users.

### Links

We provided the links to a number of other database and software in the main interface of SPTEdb (Figure 1B), including some frequently used databases for repetitive elements and some software mentioned in this work.

## Discussion

TEs are the most abundant genomic components in plants and have a major impact on the size of the plant genome. For example, the genome of *Picea abies* is very huge (around 20 Gb), but there is no evidence for the occurrence of recent whole genome duplication in it. This tremendous genome appears to be caused by a whole genome duplication in it. This tremendous genome appears to be caused by a slow and steady accumulation of various LTR retrotransposons (32). The retrotransposons are widespread and abundant in conifers. At least 20 000 copies of *Ty3/Gypsy* retrotransposons are present in the genome of *Pinus taeda*, and their total length exceeds the entire genome length of *Arabidopsis thaliana* (33). The number of DNA transposons in conifers is limited compared to the retrotransposons, probably due to the lack of effective retrotransposon elimination mechanisms in conifers (32). In addition to their numerical importance in plant genome size, TEs are now known to have a major part in genome evolution (34). Their roles include gene innovation, gene regulation, genome rearrangement (8, 35, 36) and so on. These various evolutionary implications can lead to confusion in gene annotation and can also complicate the process of genome assembly. Therefore, it is particularly crucial to annotate and classify TEs correctly in genome sequences.

There are two types of repetitive elements database at present, namely the sequences collected from diverse species or single species. Repbase, TIGR plant repeat database and PGSB-REcat are the representative of the former and have been applied widely. Nonetheless, TEs data of each species in Repbase are insufficient. For example, there are only 329 TEs of *Salicaceae* in this database. Although repetitive sequences of 11 poplar species—including *P.trichocarpa*—are recorded in PGSB-REcat, these sequences are all satellite repeats. With regard to TIGR plant repeat database, it was taken out of service due to the lack of funding on 8 February 2017. As a delegate of the second type of database, RepPop is a repetitive elements database of *P.trichocarpa*, and it contains 9623 repetitive elements, whereas only 161 of them are transposons (37). Moreover, this database does not keep up with the research of TEs on account of lacking data update.

Other databases of *Salicaceae*, such as PtGDB (http://www.plantgdb.org/PtGDB/) and PopGenIE (http://popgenie.org/), have mainly focused on genome data. We established SPTEdb under the infrastructure of the published salicaceous genome sequences. SPTEdb is dedicated to TEs identification and classification in salicaceous genomes using multiple methods to help user mining data from the TE sequences of *Salicaceae* easily and effectively. Compared with existing databases, SPTEdb provides detailed information for TEs in three salicaceous plants, and other databases can use these data to develop their specific functions. Several analysis tools were embedded in SPTEdb, such as BLAST, GetORF and HMMER, which facilitate the analysis of TEs.

Most of TEs in SPTEdb were identified from *P.trichocarpa* due to its more detailed and accurate genome information. In contrast, only small amounts of TEs were detected in *P.euphratica* and *S.suchowensis*, on account of the genome assemblies require improved. In all three species, the number of retrotransposons was greater than that of DNA transposons, probably due to the lack of retrotransposon elimination mechanisms as it in conifers. Many transposons in SPTEdb, either retrotransposons or DNA transposons, remained as singletons. This situation was more pronounced in *P.euphratica* and *S.suchowensis*, resulting in much less TEs than *P.trichocarpa*, but the difference in the number of TEs families among the three species was insignificant. For the reason of complexity and diversity of TEs, 2770 DNA transposons (*P.trichocarpa*), 200 (*P.euphratica*) and 169 (*S.suchowensis*) retrotransposons (Table 2) were not classified precisely. We will continue to collect more TEs information from other species and try to perform different analysis software, and strive for solving this issue.

Eventhough the accuracy of our method needs to be further improved, verification by *Arabidopsis thaliana* supports that our database is reliable. With increasing genome sequencing of salicaceous plants, we will continuously update and improve SPTEdb, and the submissions of new data from other researchers are encouraged.

## Conclusion

SPTEdb is a comprehensive and systemic TEs database for salicaceous plants. This database consists of 18 393 TEs from three salicaceous plants in combination with the classification information. As a user-friendly website, SPTEdb allows users not only to search, browse and download TEs data but also to analyze and compare them with the tools provided. We commit to continuously update data and improve its applications as more salicaceous genomes sequencing to be completed. Therefore, SPTEdb will contribute to the research of TEs biology in salicaceous plants and provide a platform for further study in salicaceous functional genomics.

## Funding

This work was supported by Open Foundation of State Key Laboratory of Tree Genetics and Breeding (Chinese Academy of Forestry) (TGB2016003); National Natural Science Foundation of China (31600541) and Science Foundation of China Postdoctor (160132). Funding for open access charge: National Natural Science Foundation of China.

*Conflict of interest*. None declared.
